# Peroxin 14 tags peroxisomes and interacts with Nbr1 for pexophagy in the filamentous insect pathogenic fungus *Beauveria bassiana*

**DOI:** 10.1080/27694127.2023.2168337

**Published:** 2023-01-29

**Authors:** Hai-Yan Lin, Jia-Hui Lei, Jin-Li Ding, Yue-Jin Peng, Hao Zhang, Ming-Guang Feng, Sheng-Hua Ying

**Affiliations:** Institute of Microbiology, College of Life Sciences, Zhejiang University, Hangzhou, 310058, China

**Keywords:** Pexophagy, Filamentous fungi, Insect pathogen, Vegetative growth, Stress resistance, Pathogenesis

## Abstract

Pexophagy is a main pathway for selective degradation of peroxisomes. However, the molecular mechanisms underlying pexophagy remain fragmented in the filamentous fungi. Herein, we described a complete pathway for pexophagy in the filamentous insect pathogenic fungus *Beauveria bassiana*. In *B. bassiana*, peroxin 14 (BbPex14) was a peroxisomal protein, and its loss did not completely abolish peroxisome biogenesis. The loss of BbPex14 blocked the importation of proteins with peroxisomal targeting signal type 1. Δ*Bbpex14* strain displayed the impaired phenotypes in vegetative growth, stress response, differentiation, and virulence. Notably, BbPex14 was required for pexophagy and interacted with BbNbr1 which was also involved in fungal stress response, development, and virulence. BbNbr1 sequentially interacted with autophagy-related protein 8 (Atg8) responsible for autophagosome biogenesis. BbNbr1 displayed dual association with peroxisome and autophagosome. Together, BbNbr1 act as a receptor recognizing the peroxisomal adaptor BbPex14 and translocates the targeted peroxisomes into autophagosomes. The Pex14-Nbr1-Atg8 pathway mediates pexophagy during development and stress response in *B. bassiana*. This investigation improves our understanding of the selective autophagy pathway in the filamentous fungi.

**Abbreviations:** AA: amino acid; Atg: Autophagy-related gene; BiFC: bimolecular fluorescence complementation; Co-IP: co-immunoprecipitation; GFP: green fluorescent protein; HA: hemagglutinin; LT_50_: median lethal time; Nbr1: neighbor of BRCA1 gene 1; ROS: reactive oxygen species; SAR: receptors for selective autophagy;

## Introduction

Autophagy, including microautophagy and macroautophagy, refers a conserved intracellular mechanism for recycling cellular proteins and organelles. Both types of autophagy occur in a selective or non-selective manner [1]. Peroxisomes are single membrane organelles and essentially contribute to lipid/fatty acid metabolism and detoxification of reactive oxygen species (ROS). Peroxisome homeostasis is finely maintained by biogenesis and pexophagy [2]. Peroxisome biogenesis requires a series of peroxins (Pex) [3]. Pexophagy is a selective degradation pathway which requires the receptor proteins to destine peroxisomes for autophagy [4]. The selective autophagy receptors (SAR) were firstly identified in yeasts. Autophagy-related proteins (Atg) 30 and 36 function as SARs in *Pichia pastoris* and *Saccahromyces cerevisiae*, respectively [5,6]. These two SARs associate with peroxisomes via directly binding with the peroxisome-anchored Pex3 [6,7]. Additionally, *P. pastoris* Atg30 interacts with peroxisomal membrane protein Pex14. During pexophagy, Atg30 destines the targeted peroxisomes for autophagic degradation through interacting with the scaffold protein Atg11 and the ubiquitin-like protein Atg8 [5]. Unlike unicellular yeasts, filamentous fungi develop the dense mycelia and sporulation process, which determines their unique lifestyles [8]. Thus far, the mechanistic insights involved in pexophagy remain enigmatic in filamentous fungi. No homologs of yeast Atg30 and Atg36 have been identified in filamentous fungi through by sequence alignment analyses [9]; hence, the homologs of yeast receptors are still lacking in filamentous fungi. In the filamentous ascomycete *Sordaria macrospora*, pexophagy is dependent on Nbr1 (neighbor of BRCA1 gene 1). However, how Nbr1 targets peroxisome is still unclear [10]. In mammals, Nbr1 acts as a cargo receptor for the degradation of ubiquitinated substrates via direct interaction with Atg8 [11]. In peroxisome, Pex5 and Pex7 are responsible for recognizing the matrix proteins with peroxisomal targeting signal (PTS) type 1 and 2, respectively. Pex14 is involved in formation of the docking complex which is required for importing the receptor Pex5 from cytoplasm into peroxisome [12]. In *Hansenula polymorpha* (yeast) and *Magnaporthe oryzae* (filamentous fungus), Pex14 plays critical roles in peroxisome biogenesis and pexophagy [13,14]. However, in filamentous fungi, the pathway involved in pexophagy is still in the state of fragmentation.

*Beauveria bassiana* is one of the most common species in filamentous entomopathogenic fungi and has great potential in the biological control of insect pests [15]. *B. bassiana* conidia germinate, and infective hyphae breach the host cuticle with the help of a set of hydrolytic enzymes [16]. After reaching the host hemocoel, mycelia develop into yeast-like cells (*in vivo* hyphal bodies, which facilitate fungal propagation [17,18]. At the late stage of infection, *B. bassiana* shifts pathogenic growth to saprotrophic growth and generates numerous conidia on the host cadavers for subsequent infection cycle [19]. Pexophagy participates in many physiological aspects of *B. bassiana*, including stress response, asexual sporulation and virulence [20]. Considering its unique lifestyle, *B. bassiana* represents an ideal model fungus to explore the mechanisms involved in pexophagy during fungal development and interaction with the insect hosts.

In this report, we describe that *B. bassiana* Pex14 (BbPex14) is dispensable for peroxisome biogenesis, but significantly involved in pexophagy. BbPex14 mediates recognition of the target peroxisome through direct interaction with BbNbr1 which directly interacts with the autophagic machinery Atg8. As for biological functions, the Pex14-Nbr1 pathway mediates pexophagy in fungal development and response to oxidative stress. We suggest the first complete mechanistic model for the selective degradation of peroxisomes in filamentous fungi.

## Results

### *Bioinformatic characterization of* BbPex14 *and* BbNbr1

After BLAST search, the *B. bassiana* homolog of yeast Pex14 was recognized as BBA_03432 and named as BbPex14. In addition, the *B. bassiana* homolog of *S. macrospora* Nbr1 was recognized as BBA_06072 and named as BbNbr1. As shown in Fig. S1A, the open reading frame (ORF) of BbPex14 was 1,219 bp long with three introns in genome, encoding a 348-amino acid (AA) protein. BbPex14 contained a domain of Pex14_N (PF04695.13) at amino-terminus. The BbNbr1 ORF was 4,181 bp long with three introns, encoding a 1,171-AA protein. BbNbr1 contained a series of domains, including four ZZ domains (PF00569.17) and one N_BRCA1_IG (PF16158.5). In addition, there was an Atg8-interacting motif (AIM) at carboxyl terminus.

To further determine the functions of BbPex14 and BbNbr1, two genes were individually disrupted and complemented (Fig. S1B and C).

### *The* BbPex14 *roles in peroxisomal biogenesis and biology*

**Figure 1.**
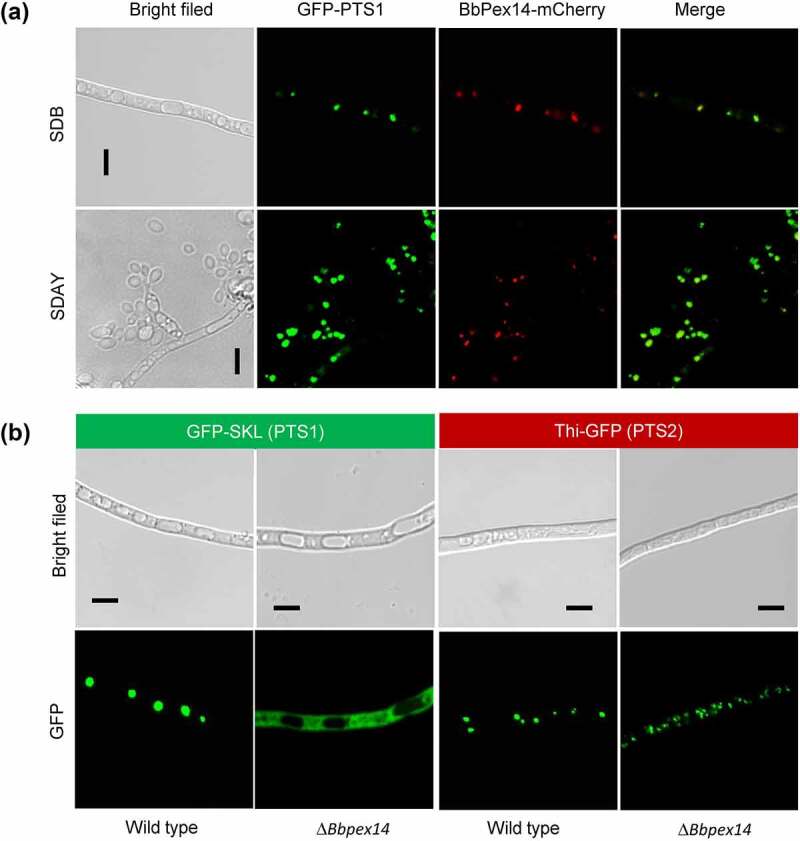
**The Pex14 roles in peroxisomal biology of *B. bassiana***. (a) Sub-cellular localization of BbPex14. *BbPEX14* was fused to *mCherry* gene and transformed into a strain in which the peroxisomes were labeled with green fluorescent protein. The transgenic fungus was cultured under submerged (SDB) and aerial (SDAY) conditions. (b) Translocation of peroxisomal proteins. The fusion proteins GFP-SKL and Thi-GFP represented the proteins with peroxisomal targeting signal (PTS) type 1 and 2, respectively. The translocation of fusion proteins was examined in the wild-type and Δ*Bbpex14* mutant strains. Fluorescent signals were detected under a laser scanning confocal microscope. Bars: 5 µm.

To view sub-cellular localization of BbPex14, its coding sequences were fused to a mCherry gene. Peroxisomes were indicated by fusing the PTS1 sequence to green fluorescent protein gene. In the transgenic strain expressing both fusion genes, globular and punctuate red signals were co-localized with the green fluorescence well (Fig. 1A). These results indicated that this peroxin localized at peroxisomes.

As shown in Fig. S2, peroxisomes were observed in the wild-type and Δ*Bbpex14* cultured in SDB medium and the medium with oleic acid as single carbon source. No significant difference was noted for peroxisomal morphology in the gene disruption mutant strain when compared with the wild-type strain. This result indicated that the BbPex14 loss did not completely abolish peroxisomal biogenesis.

Peroxisomal targeting of proteins depends on short sequences known as PTS1 and PTS2. PTS1 consists of C-terminal tripeptide (S/A/C-K/R/H-L). PTS2 is a degenerated nonapeptide (R/K-L/V/I-X_5_-H/Q-L/A) (X: random amino acid) near the N terminus [12]. A PTS2 is recognized in the peroxisomal thiolase (locus tag: BBA_04955) of *B. bassiana* and designated as BbThi [21]. The reporters GFP-SKL and BbThi-GFP were used to view the translocation activities of PTS1 and PTS2 pathways, respectively (Fig. 1B). As for GFP-SKL, granular and punctuate signals were obviously seen in the wild-type strain, but the fluorescent signals evenly distributed in cytoplasm of Δ*Bbpex14* mutant strain. As for BbThi-GFP, granular green signals were obviously observed in the wild-type and gene disruption mutant strains. These results indicated that ablation of BbPex14 affected the translocation of peroxisomal proteins with PTS1, but did not affect those with PTS2.

### *Δ*Bbpex14 *strain is impaired in growth, development, stress resistance and virulence*

Phenotypic assays were performed among the wild-type, Δ*Bbpex14*, and complemented mutant strains. Radial growth was examined on different nutrients. On carbon sources (Fig. S3A), Δ*Bbpex14* mutant strain exhibited the significant growth defects on all tested carbon sources when compared with the wild-type strain. As for nitrogen sources, the difference in colony diameter among three strains differed with nutrients. On peptone plates, no significant difference in colony diameter was observed among three strains. On the gelatin and chitin plates, Δ*Bbpex14* only displayed slight decrease in colony diameters, when compared with those of the wild-type strain.

**Figure 2.**
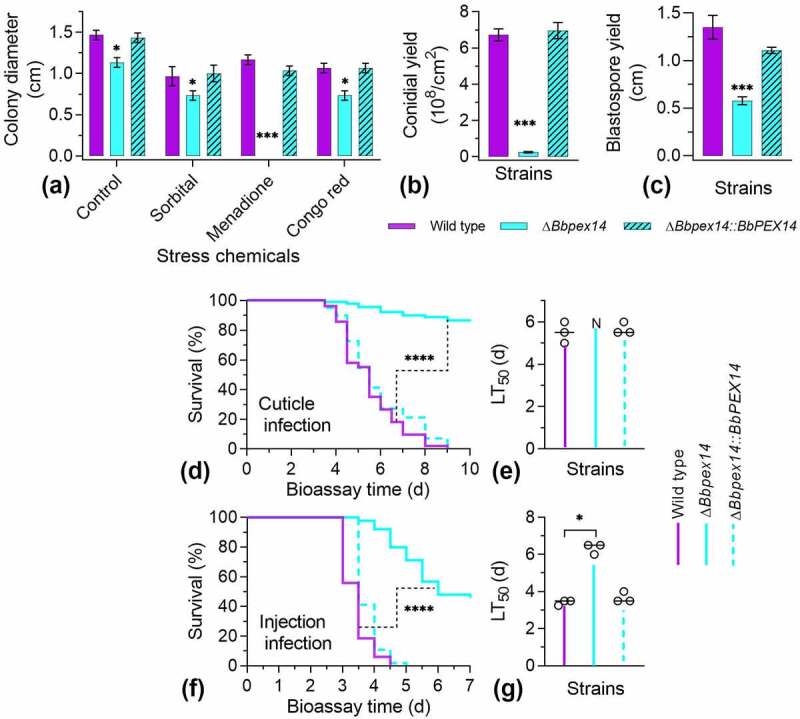
**Phenotypic assay for Δ*Bbpex14* mutant strain**. (a) Fungal stress response was determined on CZA plate included with stress chemicals. Colony diameter was examined 7 d post incubation, using CZA as control. Fungal conidiation (b) and blastospore formation (c) were quantified on SDAY plate and SDB broth, respectively. Fungal virulence was measured with two methods of cuticle (d) and injection (e) infection. Statistical significance between curves was calculated by the log-rank test. Median lethal time (LT_50_) was calculated by the Kaplan-Meier method, and the values for the cuticle and injection infection methods were shown in panel F and G, respectively. ‘N’ indicates that LT_50_ value could not be estimated due to the accumulative mortality less than 50%. Statistical significance in phenotype between the WT and gene disruption mutant strain was calculated with Student’s *t*-test. *: *P*<0.05; ***: *P*<0.001; ****: *P*<0.0001.

Under chemical stresses (Fig. 2A), there was significant difference in colony diameter among three strains under four test conditions. Under the sorbitol and Congo red stresses, gene loss resulted in a slight decrease in colony diameter, with a reduction of approximately 23% and 25%, respectively. These data were similar with that decrease in colony diameter of Δ*Bbpex14* (27%) on the control media (CZA). This implied that ablation of *BbPEX14* did not significantly influence fungal response to osmotic stress and perturbation of cell-wall synthesis in *B. bassiana*. Notably, Δ*Bbpex14* was particularly sensitive to the oxidative stress caused by menadione and did not produce obvious colony on plate.

The BbPex14 loss resulted in a dramatic decrease in conidial yield on SDAY plates (Fig. 2B). Conidial yield of Δ*Bbpex14* was 0.25 ± 0.04 × 10^8^ conidia/cm2 [mean ± standard deviation (SD)] with an approximate 95% reduction when compared with that for the wild-type strain (6.73 ± 0.33 × 10^8^ conidia/cm^2^). Under submerged condition (Fig. 2C), the wild-type strain produced 1.35 ± 0.13 × 10^8^ spores/ml. Blastospore production was significantly reduced in Δ*Bbpex14*, with approximate reduction of 57%, when compared with that of the wild-type strains. No significant difference in sporulation was observed between the wild-type and the complementation mutant strains. These data suggested that fungal differentiation was significantly impaired in Δ*Bbpex14* mutant strain.

Insect bioassays were performed by both topical infection (D) and injection (E) of the last instar larvae of the greater wax moth. In two assays, there was significant difference in the survival trend between the wild-type and disruption mutant strains (*P* < 0.0001). Notably, in topical infection assay (F), the Δ*Bbpex14* mutant strains did not result in an accumulative mortality of over 50%, which did not generate LT_50_ for the gene disruption mutant strain. In injection assay (G), statistical analysis indicated that there was significant difference in LT_50_ value between WT and Δ*Bbpex14* mutant strains. Δ*Bbpex14* exhibited an approximately doubled LT_50_ when compared with that of the WT strain. These data suggest that BbPex14 is significantly required for fungal virulence with a greater contribution to penetrating through insect cuticles.

### *Δ*Bbpex14 *strain exhibits the defects in pexophagy*

**Figure 3.**
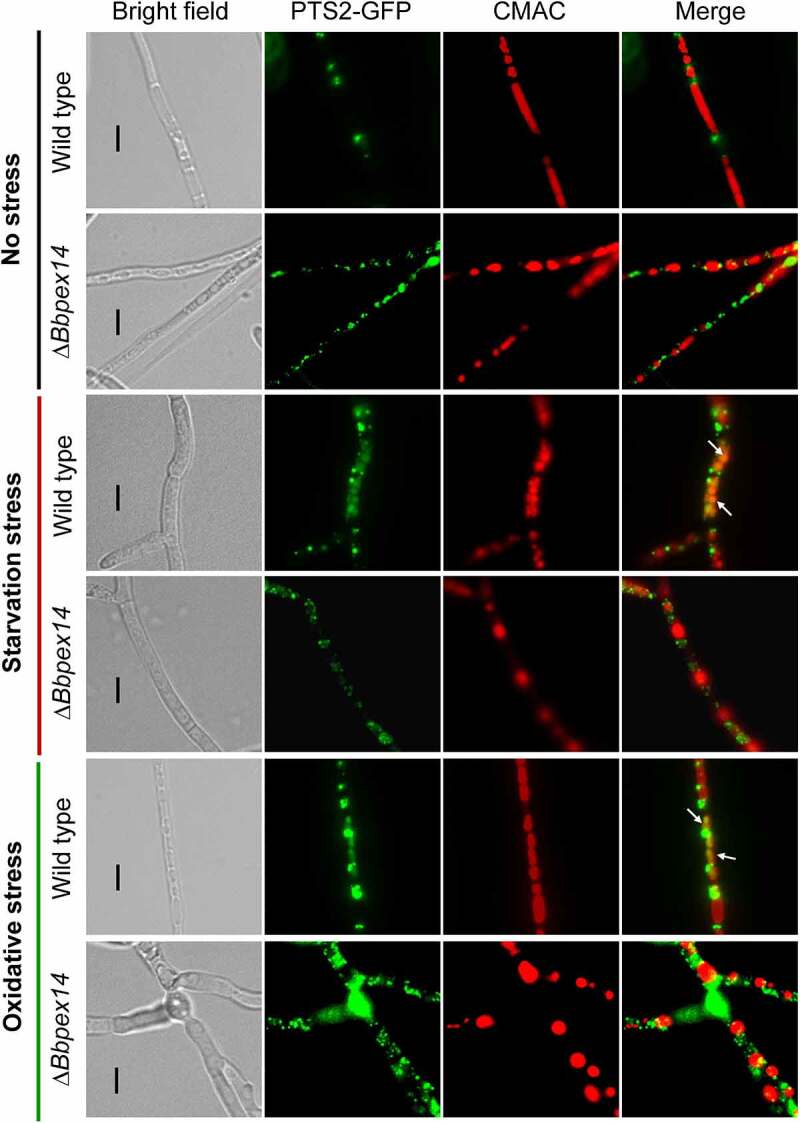
**Pex14 significantly is required for the pexophagy process of *B. bassiana***. Peroxisomes in the wild-type and Δ*Bbpex14* mutant strains were labeled with the fusion protein BbThi-GFP (PTS2-GFP), and vacuoles were indicated by fluorescence emitted from 7-amino-4-chloromethylcoumarin (CMAC). Fungal strain was grown in SDB medium under submerged condition. The resultant mycelia were subjected to the starvation and oxidative stresses. Without stress, globular and punctuate green signals were obviously observed out of the vacuoles. Under stresses, green signals were translocated into the vacuoles in the wild-type strain, but no significant signals were observed in those of Δ*Bbpex14* mutant strain. The arrows indicate the signals that appear in the vacuoles. Bars: 5 µm.

As mentioned above, ablation of *BbPEX14* did not affect the translocation process of peroxisomal proteins with PTS2. Peroxisomes were indicated by the fusion protein BbThi-GFP, and vacuoles were indicated by fluorescence emitted from 7-amino-4-chloromethylcoumarin (CMAC). In submerged mycelia, globular and punctuate green signals were obviously observed in cytoplasm and stayed out of the vacuoles. Under starvation and oxidative stresses, significant fluorescent signals were observed in the vacuoles of the wild-type strain, but not in those of Δ*Bbpex14* mutant strain (Fig. 3). These observations indicated that pexophagy could be induced by oxidative and starvation stresses, and the loss of BbPex14 blocked the pexophagy process.

### BbNbr1 *interacts with* BbPex14 *and localizes to peroxisomes*

**Figure 4.**
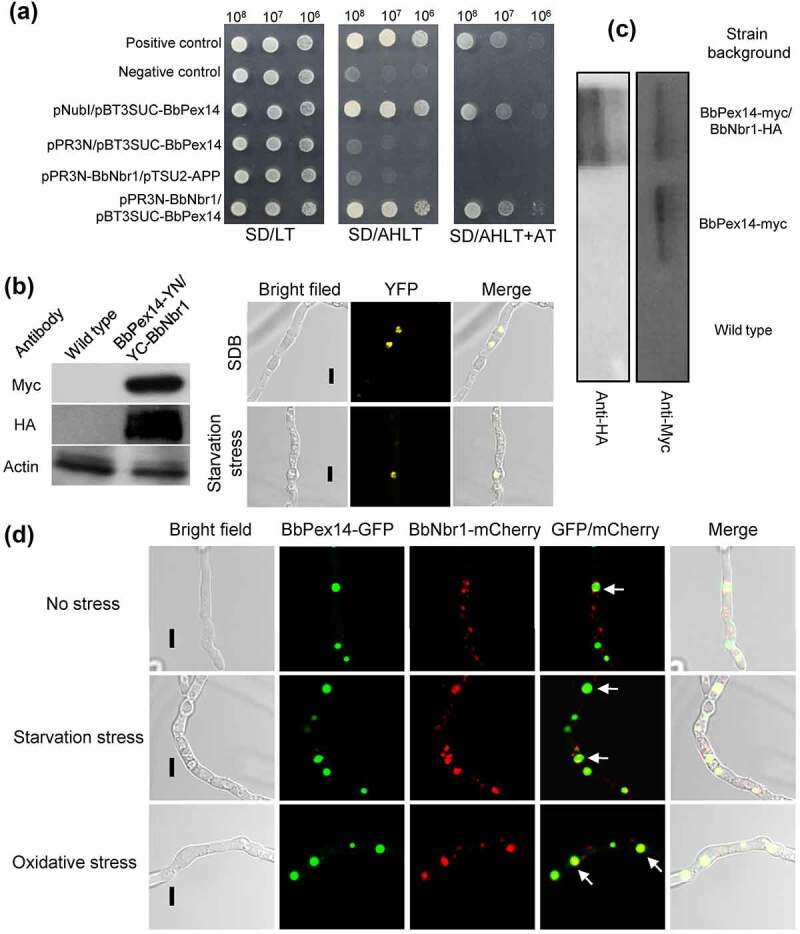
**Pex14 interacts with Nbr1 in *B. bassiana***. (a) Yeast two-hybrid (Y2H) assay. Yeast transformants were initially grown on SD/Leu-Trp (LT) medium. The protein interaction was tested on SD/Ade-His-Leu-Trp (SD/AHLT) and further confirmed on SD/AHLT plus 20 mM 3-amino-1, 2, 4-triazole (AT). Positive and negative controls were provided by kit. (b) Bimolecular fluorescence complementation (BiFC) assay. The N-terminal and C-terminal fragments of yellow fluorescent protein (YFP) (YN and YC) were fused to *BbPEX14* and *BbNBR1*, respectively. The YN and YC of the split YFP contained the coding sequence for myc and HA tag, respectively. Mycelia were cultured in SDB and stressed under starvation. The presence of their proteins was confirmed with western blot. Yellow fluorescence was seen in the mycelial cytoplasm. (c) In co-immunoprecipitation, *BbPEX14* and *BbNBR1* were fused to *Myc* and *HA*, respectively. Protein complex was precipitated with anti-myc magnetic beads, and the presence of BbNbr1 was detected with anti-HA antibody. (d) Co-localization assay. The fusion genes *BbPEX14-GFP* (representing peroxisomes) and *BbNBR1-mCherry* were transformed into the wild-type strain. Microscopy imaging revealed that most green signals co-localized with the red signals in the submerged mycelia, and this association persisted in the mycelia under the starvation and oxidative stresses. The arrows indicate the association between the red and green signals. Bar: 5 µm.

Nbr1 (neighbor of BRCA1 gene 1) mediates pexophagy in the filamentous fungus *S. macrospora* [10]. It was supposed that BbNbr1 acted as an SAR to recognize the target peroxisomes via its interaction with BbPex14 in *B. bassiana*. Pex14 is a membrane-associated protein in peroxisomes [12]. The DUALmembrane System (Dualsystems Biotech, Schlieren, Switzerland) was used to examine the interaction between BbPex14 and BbNbr1 because that this system is reliable for detecting the interaction between membrane protein and its partners. In yeast two-hybrid (Y2H) assay (Fig. 4A), all the controls worked well on the indicate plate. For instance, pNubI is the plasmid containing the wild type Nub gene and is used to test the expression of the bait gene. The transformant pNubI/pBT3SUC-BbPex14 grew well on stringent selection medium, which indicated the correct expression of *BbPEX14* in yeast. The yeast transformants with *BbPEX14* and *BbNBR1* grew well on the stringent selection medium (SD/AHLT+AT), and this result was as same as that for the strain of positive control. To demonstrate *in vivo* interaction of BbPex14 and BbNbr1, we performed a bimolecular fluorescence complementation (BiFC) assay which was based on the split yellow fluorescent protein (YFP) (Fig. 4B). The N-terminal and C-terminal fragments of YFP (YN and YC) were fused to *BbPEX14* and *BbNBR1*, respectively. Resulting expression plasmids were co-transformed into the wild-type strain of *B. bassiana*, and the presence of their proteins were validated with western blot. Significant yellow fluorescence was seen in the mycelial cytoplasm, which indicating that co-existence of BbPex14 and BbNbr1 led to the formation of a bimolecular fluorescent YFP complex. In Co-IP experiments (Fig. S4A), signals for both tags (Myc and HA) were detected in the extracts of the wild-type strain expressing the fusion genes *BbPEX14-myc* and *BbNBR1-HA*. No HA signals were detected in the wild-type strain only expressing *BbPEX14-myc*. No above signals were observed in the wild-type strain as blank control. The presence of protein in immunoprecipitates was shown in Fig. 4C, which was as same as that observed in the extracts. Together, these results indicated the paired interaction was present between BbPex14 and BbNbr1. To verify their co-localization, we performed co-transformation of the wild-type strain with the fusion genes *BbPEX14-GFP* (representing peroxisomes) and *BbNBR1-mCherry* (Fig. 4D). Fluorescence microscopy revealed that both fusion proteins produced globular and dotted signals, and most green signals co-localized with the red signals in submerged mycelia. This association pattern persisted in the mycelia subjected to oxidative and starvation stresses.

### *Δ*Bbnbr1 *strain is impaired in development, stress resistance and virulence*

**Figure 5.**
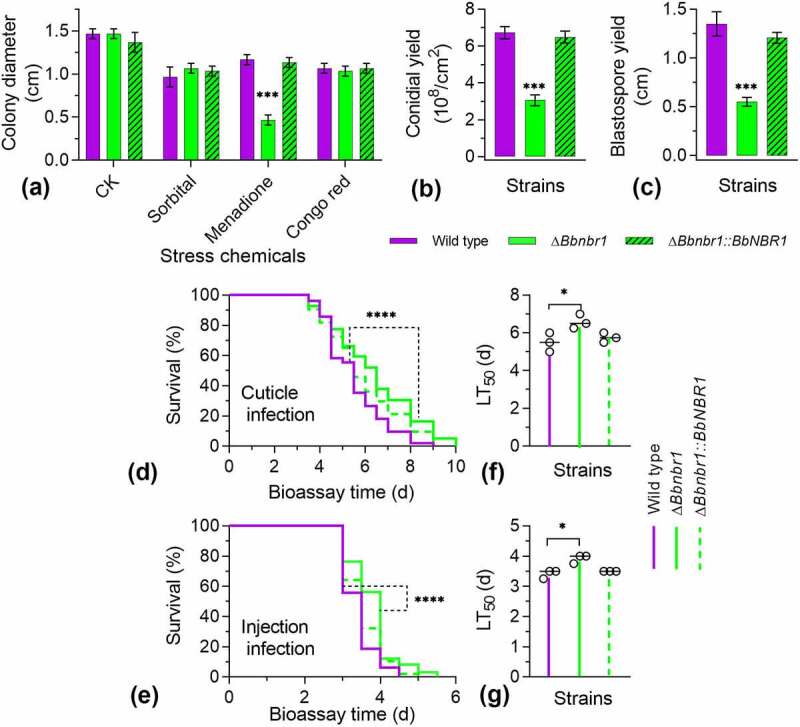
**Effects of the BbNbr1 loss on fungal phenotypes**. All phenotypic assays were performed as same as those involved in Fig. 2. Phenotypes included stress response (a), conidiation (b), and blastospore formation (c). Insect bioassay was conducted with two infection methods of cuticle application (d) and intra-hemoceol injection (e). Median lethal time (LT_50_) was calculated by the Kaplan-Meier method, and the values for the cuticle and injection infection methods were shown in panel F and G, respectively. Statistical significance between curves was calculated by the log-rank test, and other phenotypic differences between the WT and Δ*Bbnbr1* strains were determined with Student’s *t*-test. *: *P*<0.05; ***: *P*<0.001; ****: *P*<0.0001.

Phenotypic assays were performed among the wild-type, Δ*Bbnbr1*, and complemented mutant strains. Vegetative growth was examined on different nutrients (Fig. S3B). There was no significant difference in colony diameter among three strains on seven nutrients. Under chemical stresses (Fig. 5A), there was no significant difference in colony diameter among three strains under the sorbitol and Congo red stresses. Under the menadione stress, radial growth was significantly impaired in Δ*Bbnbr1* mutant strain, with a reduction of 60%, when compared with the wild-type strain (1.17 ± 0.06 cm).

Disruption of *BbNBR1* led to the severe defects in fungal sporulation on plates (Fig. 5B). On SDAY plate, Δ*Bbnbr1* mutant strain produced 3.06 ± 0.29 × 10^8^ conidia/cm^2^, with a decrease of 55%, when was compared with that for the wild-type strain. Under submerged condition (Fig. 5C), blastospore formation was also significantly impaired. The yield of Δ*Bbnbr1* was 0.55 ± 0.05 × 10^8^ spores/ml, with a decrease of 60%.

Insect bioassays indicated Δ*Bbnbr1* mutant strain displayed the weakened virulence against the insect hosts. Significant difference in survival trend was observed between the WT and Δ*Bbnbr1* mutant strains in the cuticle (D) and injection (E) infection bioassays (*P* < 0.0001). Comparison of the LT_50_ values indicated that was statistical difference between the WT and null mutant strains. Δ*Bbnbr1* displayed the LT_50_ delay of approximately 20% and 15% in the cuticle (F) and injection (G) infection bioassays, respectively. These data suggest that BbNbr1 plays a slight contribution to fungal virulence with more influence in penetrating insect cuticles.

### *Δ*Bbnbr1 *mutant strain exhibits the defects in pexophagy*

**Figure 6.**
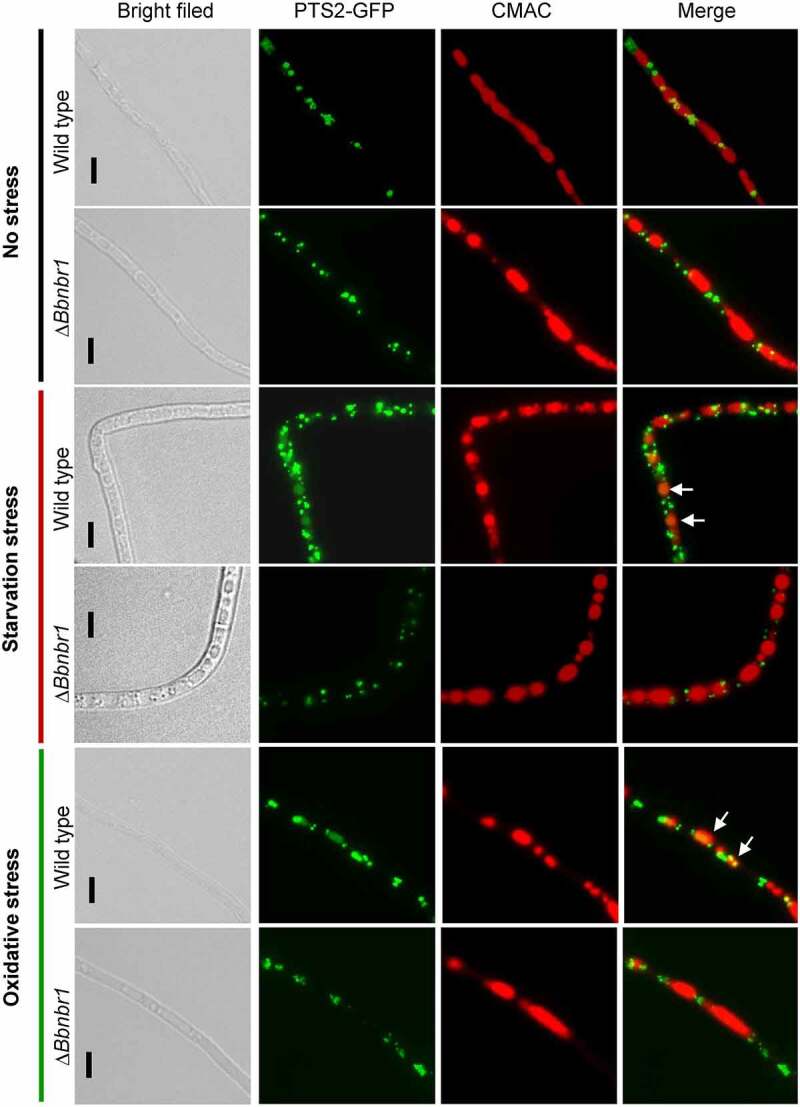
**The Nbr1 role involved in the pexophagy process of *B. bassiana***. Autophagosomes were labeled with the fusion protein GFP-Atg8, and vacuoles were indicated by fluorescence emitted from 7-amino-4-chloromethylcoumarin (CMAC). Mycelia were cultured in SDB medium and exposed the starvation and oxidative stresses. Without stress, globular and punctuate green signals were evenly distributed in cytoplasm. Upon under stresses, green signals appeared in the vacuoles of the wild-type strain, but no significant signals were observed in those of the gene disruption mutant strain. The arrows indicate the signals that appear in the vacuoles. Bars: 5 µm.

As shown in Fig. 6, peroxisomes were indicated by the fusion protein BbThi-GFP, and vacuoles were indicated by fluorescence emitted from CMAC. To make the fluorescent signals more clear, the CMAC signals was shown in red. In submerged mycelia, globular and punctuate green signals were obviously observed in cytoplasm, and no significant signal was seen in the vacuoles of the WT and Δ*Bbnbr1* mutant strains. Under starvation and oxidative stresses, fluorescent signals were translocated into the vacuoles of the WT strain, and no significant signal appeared in those of Δ*Bbnbr1* mutant strain. These data indicated that the loss of BbNbr1 significantly impaired the pexophagy under stress.

### BbNbr1 *interacts with* BbAtg8 *and associates with autophagosome-like structures*

**Figure 7.**
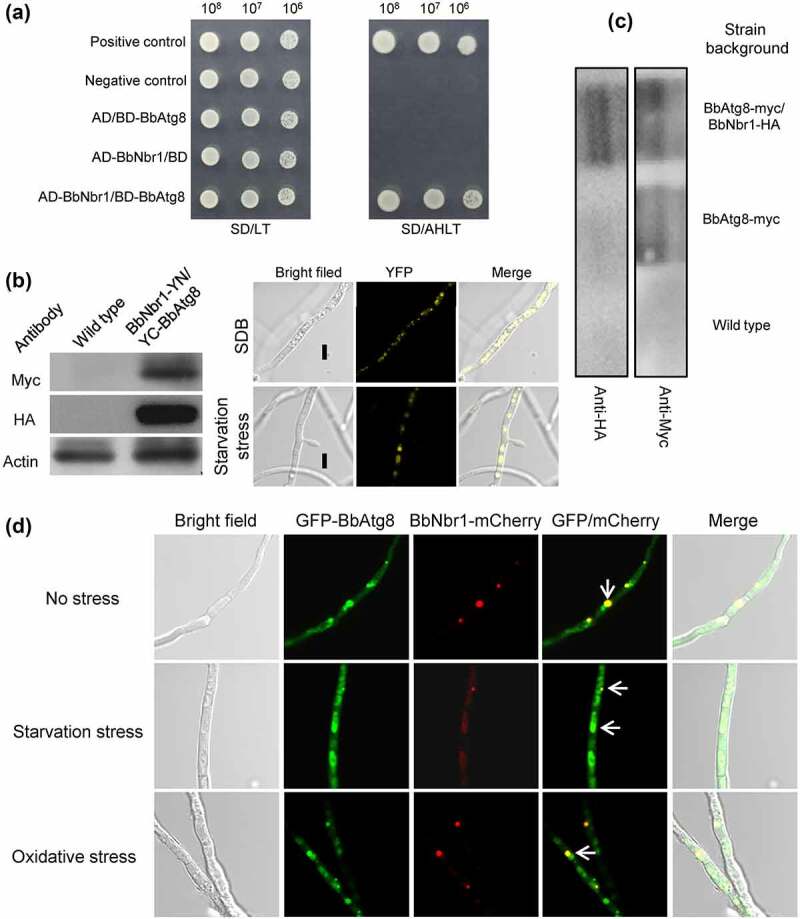
**Nbr1 is associated with autophagosomes in *B. bassiana***. (a) Yeast two-hybrid (Y2H) assay was used to test the interaction between BbAtg8 and BbNbr1. Yeast strains were initially grown on SD/Leu-Trp (LT) medium and confirmed on SD/Ade-His-Leu-Trp (SD/AHLT). Positive and negative controls were provided by kit. Methods for bimolecular fluorescence complementation (b) and co-immunoprecipitation (co-IP) assays (c) were as same as those in Fig. 4. The N-terminal and C-terminal fragments of yellow fluorescent protein (YFP) (YN and YC) were fused to *BbNBR1* and *BbATG8*, respectively. In co-IP, *BbATG8* and *BbNBR1* were fused to *Myc* and *HA*, respectively. (d) In co-localization experiment, the fusion genes *GFP-BbATG8* (representing autophagosomes) and *BbNBR1-mCherry* were transformed into the wild-type strain. Microscopy imaging revealed that the red signals co-localized with the green signals in the stressed and non-stressed mycelia. The arrows indicate the association between the red and green signals. Bar: 5 µm.

As revealed above, BbNbr1 contained an AIM at C-terminus. Matchmaker® Gold Y2H System (Clontech Laboratories, CA) was firstly used to determine the interaction between BbNbr1 and BbAtg8 owing to its suitability in detecting the interaction between soluble proteins. BbAtg8 was regarded as a bait, and BbNbr1 as a prey. All controls worked well on the indicated medium. The yeast cells expressing *BbATG8* and *BbNBR1* grew well on the selection plate (SD/AHLT), and this result was as same as that for the strain as the positive control (Fig. 7A). To demonstrate *in vivo* interaction between two proteins, we performed a BiFC assay based on the split YFP (Fig. 7B). *BbNBR1* and *BbATG8* were fused to YN- and YC- fragments of YFP, respectively. The expression plasmids were co-transformed into the wild-type strain of *B. bassiana*, and their proteins were confirmed by western blot analyses. Yellow fluorescent signals were obviously observed in mycelia, which indicating that BbAtg8 and BbNbr1 generated a bimolecular fluorescent YFP complex. In Co-IP assay (Fig. S4B), the Myc- and HA-tagged proteins were detected in the extracts from the wild-type strain with the fusion genes *BbATG8-myc* and *BbNBR1-HA*. No HA signals were detected in the wild-type strain only expressing *BbATG8-myc*. Both above signals were not detected in the wild-type strain. After immunoprecipitation, the proteins in immunoprecipitates were examined with western bolt analyses, and their presences were as same as those observed in the cell lysates (Fig. 7C). These results indicated that BbNbr1 interacted with BbAtg8. To view their co-localization in mycelia (Fig. 7D), we performed co-transformation of the wild-type strain with the fusion genes *BbATG8-GFP* (representing autophagosomes) and *BbNBR1-mCherry*. Fluorescence assay indicated that both fusion proteins produced globular and dotted signals, and most red signals co-localized with the green signals in submerged mycelia. This association pattern could also be observed in the mycelia under the oxidative and starvation stresses.

## Discussion

Peroxisome is highly dynamic organelle, and its degradation is dependent on the process of pexophagy [13]. To date, the pathway for pexophagy remains fragmented in filamentous fungi. In this study, we deciphered a complete molecular pathway underlying the selective degradation of peroxisomes in a filamentous insect pathogenic fungus *B. bassiana*.

In peroxisome, Pex14 is a component of the receptor-docking complex at the organellar membrane which is required for protein importation from cytoplasm into peroxisomal lumen [12]. In *P. pastoris*, Pex14 is absolutely required for importation of PTS1 and PTS2 proteins [22]. In *B. bassiana*, BbPex14, associated with peroxisomes, is dispensable for peroxisome biogenesis, but indispensable for peroxisomal targeting of PTS1 proteins. As for biological function, BbPex14 only partially contributes to the fatty-acid assimilation. In *B. bassiana*, BbPex5, the receptor for PTS1 proteins, is necessary for uptake of fatty acids [21]. These findings suggest that there might be a functionally alternative of Pex14 involved in the translocation of PTS1 proteins in *B. bassiana*. In addition, BbPex14 is required for response to oxidative stress, asexual development, and virulence. The oxidative stress occurs in the *B. bassiana*–host interaction, and stress tolerance is critical for fungal virulence [23]. In the host hemocoel, *B. bassiana* undergoes dimorphic change and develops into yeast-like hyphal bodies (*in vivo* blastospore), which facilitates fungal propagation in the host [18]. *B. bassiana* employs conidiation to produce numerous conidia, which promotes fungal dispersal and potential to cause subsequent infection cycle [19]. The defects in growth on fatty acid, oxidation tolerance, and dimorphism contribute to the attenuated virulence and dispersal ability of Δ*Bbpex14* strain. In *M. oryzae*, Pex14 is indispensable for peroxisomal biogenesis and plays the important roles in degradation of fatty acids, stress resistance, development, and pathogenicity [24]. Similar roles of Pex14 are also present in *Fusarium graminearum* (a plant pathogenic fungus), with additional contribution to the biosynthesis of mycotoxins [25]. These evidences indicate that Pex14 homologs perform the divergent roles in fungal species.

In *B. bassiana*, pexophagy has been linked to fungal development, oxidation resistance and virulence [18]. Herein, our results indicate that BbPex14 is significantly required for pexophagy in *B. bassiana*. Similarly, Pex14 is critical for pexophagy in a filamentous mycopathogen *M. oryzae* [14]. In yeasts of *H. polymorpha* and *P. pastoris*, Pex14 is indispensable for the selective autophagy of peroxisomes [5,13]. Hence, BbPex14 acts as a peroxisomal adaptor owing to its localization to the docking complex in organellar membrane. In mammalian cells, Pex14 plays dual roles in peroxisomal biogenesis and degradation [26]. However, in *F. graminearum*, Pex14 plays an opposite function in pexophagy. Its loss results in the enhanced pexophagy [25]. These findings suggest that the contributions of Pex14 homologs to pexophagy are likely conserved in organisms, but in different manners.

In pexophagy, the recognition of organelle is mediated by the receptors for selective autophagy (SAR). SARs in yeasts of *P. pastoris* and *S. cerevisiae* are Atg30 and Atg36, respectively [5,6]. Both two receptors recognize Pex3 in peroxisomal membrane via direct interaction [6,13]. Additionally, Atg30 binds to other peroxisomal proteins such as Pex14 and Atg37 [5,27]. No homolog of Atg30 and Atg36 has been identified in filamentous fungi through bioinformatic analyses [9]. In filamentous fungi, the first SAR indentified for pexophagy is Nbr1 of *S. macrospora* [10], and its homolog in mammal functions as a specific receptor in pexophagy [28]. In this study, the Nbr1 ortholog of *B. bassiana* is also involved in pexophagy. Notably, Pex14 is highlighted as the recognition target of BbNbr1 via direct interaction. Co-localization assay indicated that BbNbr1 is associated with peroxisomes during fungal growth, development, and stress response. This indicates that Nbr1 is also a membrane-associated receptor which is similar with Atg30 and Atg36 [5,6]. Nbr1 and Atg30 do not share significant sequence similarity, but they execute the same function in recognizing peroxisomes. These lines of evidences imply that these receptors might have structural similarity for target recognition, although the more investigations are needed. In *B. bassiana*, BbNbr1 contributes to oxidation tolerance, development, and virulence. Similarly, the loss of Nbr1 leads to defects in vegetative growth under starvation and sexual development in *S. macrospora* [10]. However, Pex14 plays a more predominant role than Nbr1 in *B. bassiana*. This result suggests that the overlapped roles of Nbr1 and Pex14 might attribute to pexophagy, and the additional roles of Pex14 are related to its peroxisomal biology.

Atg8, a ubiquitin-like protein, is not only essential for autophagosome biogenesis but also an important anchor protein in the selective autophagy via direct interaction with the SARs [29]. In *S. macrospora*, the cargo receptor Nbr1 binds directly to Atg8 during the selective pexophagy [10]. In *B. bassiana*, BbNbr1 has an AIM and also directly binds with BbAtg8. Co-localization assay indicated that BbNbr1 is associated with autophagosomes during fungal growth, development, and stress response. Similar evidences are also present in mammal and plant cells [30,31]. These suggest that the targeting of substrates into autophagy by the Nbr1 homologs is conserved in many eukaryotes. In yeast, Atg11 acts as a scaffold protein that mediates pexophagy through interacting with SARs (Atg30 and Atg36) [32]. In *P. pastoris*, Atg30 must interact with both Atg8 and Atg11 for pexophagy independently and not simultaneously [33]. In *B. bassiana*, Atg11 is also required for the selective degradation peroxisomes [20]. This suggests that there might be a similar interaction pattern for the receptor Nbr1 in *B. bassiana*. In mammals, Pex14 directly interacts with LC3-II (the mammalian homolog of yeast Atg8) to initiate pexophagy under the *in vivo* starvation condition [26]. These findings indicate that the pexophagy pathways are divergent among different organisms.

In conclusion, *B. bassiana* Pex14, a peroxisomal protein, is dispensable for peroxisome biogenesis and plays an important role in the import of PTS1 proteins. Pex14 contributes to fungal vegetative growth, stress response, differentiation, and virulence. Significantly, Pex14 acts as an adaptor and interacts with the receptor BbNbr1 to mediate pexophagy during fungal growth and response to oxidative and starvation stresses. The receptor Nbr1 translocates the targeted peroxisomes into autophagosomes via direct interaction with Atg8. This investigation provides a complete pathway for pexophagy in the entomopathogenic fungi and facilitates our understandings of pexophagy in the filamentous fungi.

## Materials and methods

### Strains and growth conditions

As described previously [20], the wild type of *B. bassiana* ARSEF2860 was used as a parent strain and cultivated Sabouraud dextrose agar (SDAY: 4% glucose, 1% peptone, 1.5% agar, and 1% yeast extract). *Escherichia coli* DH5α (Invitrogen, Carlsbad, CA, USA) was used for amplification of recombinant plasmids and cultured in Luria-Bertani (LB) medium. Clone selection was conducted by the addition of necessary antibiotics. *Agrobacterium tumefaciens* AGL-1 was used as donor strain in fungal transformation and grown in yeast extract broth (w/v: 0.5% sucrose, 1% protein, 0.1% yeast extract, 0.05% MgSO_4_, and 1.5% agar). Czapek-Dox agar (CZA) (3% sucrose, 0.3% NaNO_3_, 0.1% K_2_HPO_4_, 0.05% KCl, 0.05% MgSO_4_, and 0.001% FeSO_4_ plus 1.5% agar) was used as defined medium in assays for screening transformant and phenotypic determination.

### *Molecular identification of* B. bassiana *Pex14 and Nbr1*

For bioinformatic analyses, BLAST program was used to search the potential Pex14 and Nbr1 in the *B. bassiana* genome [34] using *S. cerevisiae* Pex14 (GenBank No.: AAS56829) and *S. macrospora* Nbr1 as query sequence, respectively. Domain annotation was conducted on the online portal SMART (http://smart.embl-heidelberg.de/) [35].

Sub-cellular localizations of BbPex14 determined as described previously [20]. All primers were shown in Table S1. Firstly, gene mCherry was fused to the downstream of coding sequence for BbPex14, and the hybrid gene was cloned into plasmid pBMRS [36]. This recombinant plasmid was transformed into the wild-type strain with peroxisomes labeled with green fluorescent protein [20]. Fluorescent signals were detected in aerial and submerged mycelia. Aerial mycelia were prepared by cultivating fungal strain on SDAY plates at 25°C for 5 d. Submerged mycelia were obtained by culturing fungal strain in SDB medium (SDAY plate without agar) at 25 °C for 2 d. The resultant samples were examined under a laser scanning confocal microscope (LSCM) (LSM 710, Carl Zeiss Microscopy GmbH, Jena, Germany).

### Molecular manipulation for gene disruption and complementation

All primers used in this study are given in Table S1. Gene disruption mutant was constructed with the fluorescence-coupled double screening method. The complemented mutant was reconstituted by ectopically integrating the whole gene into the genome of the gene disruption mutant strain [36]. The up- and down-stream fragments of the indicated gene were amplified by primer P_X_1/P_X_2 and P_X_3/P_X_4 (X: *BbPEX14* and *BbNBR1*), respectively, and sequentially recombined in the *Xma*I/*Bam*HI sites and *Xba*I/*Spe*I sites in plasmid p0380-GTB, in which bar cassette confers phosphinothricin resistance. The resultant plasmid was named p0380-X-KO, and then transformed in the wild-type strain for gene disruption. The transformants were grown on CZA plates with phosphinothricin (200 µg/ml) (45520, Sigma, MO, USA). The candidate disruptants were screened by PCR reaction with primer P_X_5 and P_X_6, and was further confirmed when no fluorescence was observed under a LSCM. To reconstitute the complementation strain, the entire gene with its promoter region, was amplified with the primer P_X_7 and P_X_8. The obtained fragment was cloned into the vector p0380-sur-gateway with chlorsulfuron resistance gene (*sur*). The resulting plasmid p0380-sur-X was transformed into the gene disruption mutant. Transformants were cultivated on CZA plates with chlorsulfuron (C11325000, Dr. Ehrenstorfer GmbH, Augsburg, Germany) and verified by PCR with the primer pair P_X_5 and P_X_6.

### *Effects of the* BbPEX14 *loss on peroxisomal biology*

Transmission electron microscopy (TEM) was used to determine peroxisomal morphology and conducted as described previously [22]. Briefly, the 2-day old mycelia were grown in SDB and the oleic-acid medium (0.3%), respectively. The resultant mycelia were washed with sterile water and fixed in K_2_MnO_4_ solution (*w/v*: 1.5%) for 20 min at 20°C. Then, the mycelia were dehydrated and embedded in resin. Ultrathin sections were stained and examined under a transmission electron microscope (Model H-7650, Hitachi).

Translocation of peroxisomal proteins into peroxisomes is dependent on the peroxisomal targeting signal (PTS) type 1 and type 2. Targeting process of peroxisomal proteins was determined as described previously [22]. In brief, the plasmids pBMS-GFP-PTS1 [20] and pBMGS-BbThi-GFP [22] generated GFP with PTS1 at C-terminus and GFP with PTS2 at N-terminus, respectively. These two plasmids were transformed into the wild-type and Δ*Bbpex14* mutant strains. Mycelia were grown in SDB medium for 2 d at 25°C and fluorescent signals were examined under a LSCM.

### Assays for vegetative growth, stress response and asexual development

Fungal strains were cultured on SDAY at 25°C for 7 d, and conidia were used as initial inocula in phenotypic assays which were conducted as described previously [36].

Vegetative growth was assessed by measurement of the colony diameter on the modified CZA plates incubated at 25°C for 7 d. Carbon sources in CZA (final concentration) were replaced with glucose (3%), sucrose (3%), trehalose (0.3%), and oleic acid (0.3%). Nitrogen sources (final concentration) included gelatin (0.5%), peptone (0.5%), and chitin (0.5%).

Fungal stress response was evaluated by measurement of the colony diameter on the chemical-supplemented CZA plates incubated at 25°C for 7 d. Chemical stressors (final concentration) included sorbitol (1 M), menadione (0.02 mM), and Congo red (3 μg/ml). CZA plate without stressor was used as a control.

Sporulation was assessed on SDAY plate (conidiation) and in SDB medium (blastospore formation). Conidial production was quantified by harvesting conidia from the 8-d-old mycelial disc and shown as conidial number per square centimeter. Blastospore yield was quantified by determining the spore concentration in 3-d-old broth and shown as spore number per milliliter.

Two inoculation methods were used to examine conidial virulence in insect model, in which 30-35 larvae of *Galleria mellonella* were used as a treatment. In topical inoculation method, larvae were flooded with conidial suspension (10^7^ conidia/ml) for 10s. In the intrahemocoel infection assay, 5-µl conidial suspension (10^5^ conidia/ml) was injected into the insect hemocoel. Tween-80 solution (0.02%) was used as blank control in two methods. The infected larvae were reared at 25°C, and the mortality was recorded daily. The median lethal time (LT_50_) was calculated by Kaplan-Meier method with log-rank test.

### Visualization of the vacuolar targeting of peroxisomes

To view effects of the BbPex14 loss on the vacuolar targeting of peroxisomes, plasmid pBMGS-BbThi-GFP [22] was transformed into the wild-type and Δ*Bbpex14* mutant strains. The transgenic strains were grown in SDB medium at 25°C for 2 d. The resulting mycelia were divided into three parts. One part was transferred into fresh SDB (no stress). The second part was transferred into fresh SDB plus 2 mM menadione for oxidative stress. The third part was washed and transferred into Czapek-Dox medium without carbon and nitrogen sources for starvation stress. All treatments were incubated for three more hours. Fluorescent dye FM4-64 was used to indicate vacuoles. Fluorescent signals in mycelia were examined under a LSCM.

To view effects of the BbNbr1 loss on the vacuolar targeting of peroxisomes, 7-amino-4-chloromethylcoumarin (CMAC) was used to indicate vacuoles.

### Protein interaction between BbNbr1 and BbPex14

All primers were listed in Table S1. The interaction between BbPex14 and BbNbr1 was firstly determined in the DUALmembrane system (Dualsystems Biotech, Schlieren, Switzerland). The prey vector pPR3-N, infused with BbNBR1, and the bait vector pBT3-SUC-BbPEX14 were co-transformed pairwise into the yeast strain NMY51. Yeast cells were SD/Leu-Trp medium. The protein interaction was preliminarily determined when the transformants on SD/Ade-His-Leu-Trp (SD/AHLT) and further confirmed on SD/AHLT plus 3-amino-1, 2, 4-triazole (20 mM). Positive and negative controls were provided by kit.

Bimolecular fluorescence complementation (BiFC) assay was conducted as described previously [37]. *BbPEX14* and *BbNBR1*were cloned into expression vectors p0380-TEF-MCS-YN-sur and p0380-TEF-MCS-YC-bar, respectively. The resulting vectors were successively transformed into the wild-type strain. The transformant was cultured in SDB medium and the presence of YN-BbPex14 and YC-BbNbr1 was detected with immunoblotting assay, using anti-myc and anti-HA antibody, respectively. Actin was used as a reference. YFP signals in mycelia were examined under a LSCM.

For co-immunoprecipitation (Co-IP) experiment, *BbPEX14* and *BbNBR1* were tagged with the coding sequences for Myc and HA, respectively. Two fusion genes were cloned into expression vectors p0380-TEF-MCS-TER-bar and p0380-TEF-MCS-TER-sur, respectively. The resultant vectors were transformed into the wild-type strain. The resultant transgenic strain was cultured in SDB. Mycelia were sampled and ground in liquid nitrogen. The protein extracts were prepared in PBS buffer. Anti-myc magnetic beads (P2118, Beyotime Biotechnology, Shanghai, China) were incubated with protein sample at 25°C for 1 h. The captured proteins were eluted and resolved in SDS-PAGE. The presence of BbPex14 and BbNbr1 was detected with immunoblotting assay, using anti-myc and anti-HA antibody. The wild-type and a strain with *BbPEX14-Myc* were used as blank and expression controls, respectively.

To view co-localization of BbPex14 and BbNbr1, their genes were cloned into plasmids pBMGB [38] and pBMRS [36], respectively. Two plasmids were transformed into the wild-type strain. The transformants were cultured in SDB at 25°C for 2 d, and the resulting mycelia were further subjected to starvation and oxidative stresses as mentioned above. Dual fluorescence analysis was conducted under an LSCM.

### Protein interaction between BbNbr1 and BbAtg8

There was an Atg8-interacting motif (AIM) at carboxyl terminus of BbNbr1. Their interaction was firstly verified in Matchmaker® GAL4 Two-Hybrid System 3 kit (Clontech Laboratories, CA). In brief, *BbNBR1* and *BbATG8* were cloned into the vectors pGADT7 and pGBKT7, respectively. These two plasmids were co-transformed into yeast YH109 and transformants were validated on SD/Leu-Trp plate. The protein interaction was determined when the transformants grew well on SD/Trp-Leu-His-Ade medium.

To further establish the interaction between these two proteins, BiFC and Co-IP were conducted as same as those methods used in detecting the interaction of BbNbr1 with BbPex14. Co-localization of BbAtg8 and BbNbr1 was established with dual fluorescence analysis as same as those used to view co-localization of BbPex14 and BbNbr1. All primers were listed in Table S1.

### Statistical analysis

All phenotypic measurements for the wild-type, gene disruption and complementation strains were subjected to Student’s *t*-test, and the significance was determined when *P* is less than 0.05.

## Supplementary Material

Supplemental Material

## Data and materials availability

All data are available in the main text or the supplementary materials.
