# Supplementary material for: Peroxin 14 tags peroxisomes and interacts with Nbr1 for pexophagy in the filamentous insect pathogenic fungus Beauveria bassiana
Source: Autophagy Rep. 2023 Jan 29;2(1):2168337. doi: 10.1080/27694127.2023.2168337 (PMC12042473; doi:10.1080/27694127.2023.2168337)

**Table S1. Primers for molecular manipulation in *Beauveria bassiana***

| **Primers** | **Paired sequences (5′-3′)*** | **Purpose** |
| --- | --- | --- |
| ***PEX14*** | | |
| P_X_1/P_X_2 | TGGGCCCGGCGCGCCGAATTCTGGCAAGGTGGTTGTAGT | Amplifying upstream flanking sequence |
|  | TGGCTGCAGGTCGACGGATCCAGAAAGATGACGAGGGAAC |  |
| P_X_3/P_X_4 | GACCCATGGCTCGAGTCTAGAGCGAGCATTCACCCTTGT | Amplifying downstream flanking sequence |
|  | GGTGGTGGTGGCTAGCGTTAACACGTACTGCGACTGCTCC |  |
| P_X_5/P_X_6 | ATACCGAACGCAGCACAT | Screening the candidate mutants |
|  | CAAGCACGCACTAACCAC |  |
| P_X_7/P_X_8 | ATCCGTCGACCTGCAGCCAAGCTTGCTCATCAGCCACGAAT | Cloning the entire gene |
|  | ACACTAGTCAGATCTTCTAGTGTGCTCCCAGTGACCCTTT |  |
| PL_X_1/PL_X_2 | CAATCACAAACACCTTCAAAATGGCGATTCGCGAGGACCTAGTGGC | Intracellular localization |
|  | CTCGCCCTTGCTCACCATAGAACTGCTGGAAGCATCCTCGCCGGTA |  |
| PY_X_1/PY_X_2 | CCAAAATATCTGCAATGGCCATGGCGATTCGCGAGGACCTAGTGGC | Construction of bait plasmid for yeast two-hybrid (Y2H) |
|  | TCGAATTCCTGCAGATGGCCAGAACTGCTGGAAGCATCCTCGCCGGTA |  |
| PB_X_1PB_X_2 | TCGCATTCAATCACAAACACCTTCAAAATGGCGATTCGCGAGGACCTAGTGGC | For BiFC (YN) |
|  | CAACTTTTGCTCCATGTCGACGGATCCAGAACTGCTGGAAGCATCCTCGCCGGTA |  |
| ***NBR1*** | | |
| P_X_1/P_X_2 | AGCTGTACAAGTAACCCGGGCTGTGAGCAGCGAGAAGA | Amplifying upstream flanking sequence |
|  | GGCTGCAGGTCGACGGATCCTGACTATTTGATGAGCGTGT |  |
| P_X_3/P_X_4 | GACCCATGGCTCGAGTCTAGATGCCAGAAATGCGATGAT | Amplifying downstream flanking sequence |
|  | GGTGGTGGTGGCTAGCGTTAACCGTGCTGCAACGACCTAC |  |
| P_X_5/P_X_6 | TTCATCCATCCGTTTCATC | Screening the candidate mutants |
|  | TGGTCAGGCATCAGAGTG |  |
| P_X_7/P_X_8 | ATCCGTCGACCTGCAGCCAAGCTTTAGGCAAGGTAAGAAGATGA | Cloning the entire gene |
|  | ACACTAGTCAGATCTTCTAGTGTGGAGGCACTGGGTCAT |  |
| PY_X_1/PY_X_2 | TCAACGCAGAGTGGCCATGTCGGCTGCTCCTACTG | Construction of Prey plasmid for Y2H (DUAL membrane system) |
|  | CTCGAGAGGCCGAGGCGGCCTTAGTTAACATTATTGGTCAGCGAG |  |
| PY_X_3/PY_X_4 | GCCATGGAGGCCAGTGAATTCATGTCGGCTGCTCCTACTGCTC | Construction of prey plasmid for Y2H  (nuclear system) |
|  | CAGCTCGAGCTCGATGGATCCTTAGTTAACATTATTGGTCAGCGAGGG |  |
| PB_X_1PB_X_2 | TCGCATTCAATCACAAACACCTTCAAAATGTCGGCTGCTCCTACTGCTC | For BiFC (YC) |
|  | GGGTACATCTGCAGGTCGACGTTAACATTATTGGTCAGCGAGGGC |  |
| PB_X_3PB_X_4 | TCGCATTCAATCACAAACACCTTCAAAATGTCGGCTGCTCCTACTGCTC | For BiFC (YN) |
|  | CAACTTTTGCTCCATGTCGACGGATCCGTTAACATTATTGGTCAGCGAGGGC |  |
| ***ATG8*** | | |
| PY_X_3/PY_X_4 | ATGGCCATGGAGGCCGAATTCATGCGTAGCAAGTTCAAGGATGAGC | Construction of bait plasmid for yeast Y2H (nuclear system) |
|  | CGCTGCAGGTCGACGGATCCTCAAATGCTGCCAAAGGTGTTCTCG |  |
| PB_X_1PB_X_2 | ATACGATGTTCCAGATTACGCTCCCGGGATGCGTAGCAAGTTCAAGGATGAGC | For BiFC (YC) |
|  | CTCGGTACCAAGCTTGGCTGCAGGTCGACTCAAATGCTGCCAAAGGTGTTCTC |  |

*: The underlined region in the primer was required for homologous recombination in plasmid preparation.

**Figure S1. Domain analyses and molecular manipulation of *B. bassiana* Pex14 and Nbr1.** (A) Domain architecture of BbPex14 and BbNbr1. (B) PCR analysis to screen the gene disruption and complemented mutant strains. Lane1: wild type, lane 2: disruption mutant, lane 3: complementation strain, lane M: DNA marker. (C) Representative images for homologous and non-homologous recombination events. The fluorescent signals were examined under a laser scanning confocal microscope. The wild type was used as control of no fluorescence.


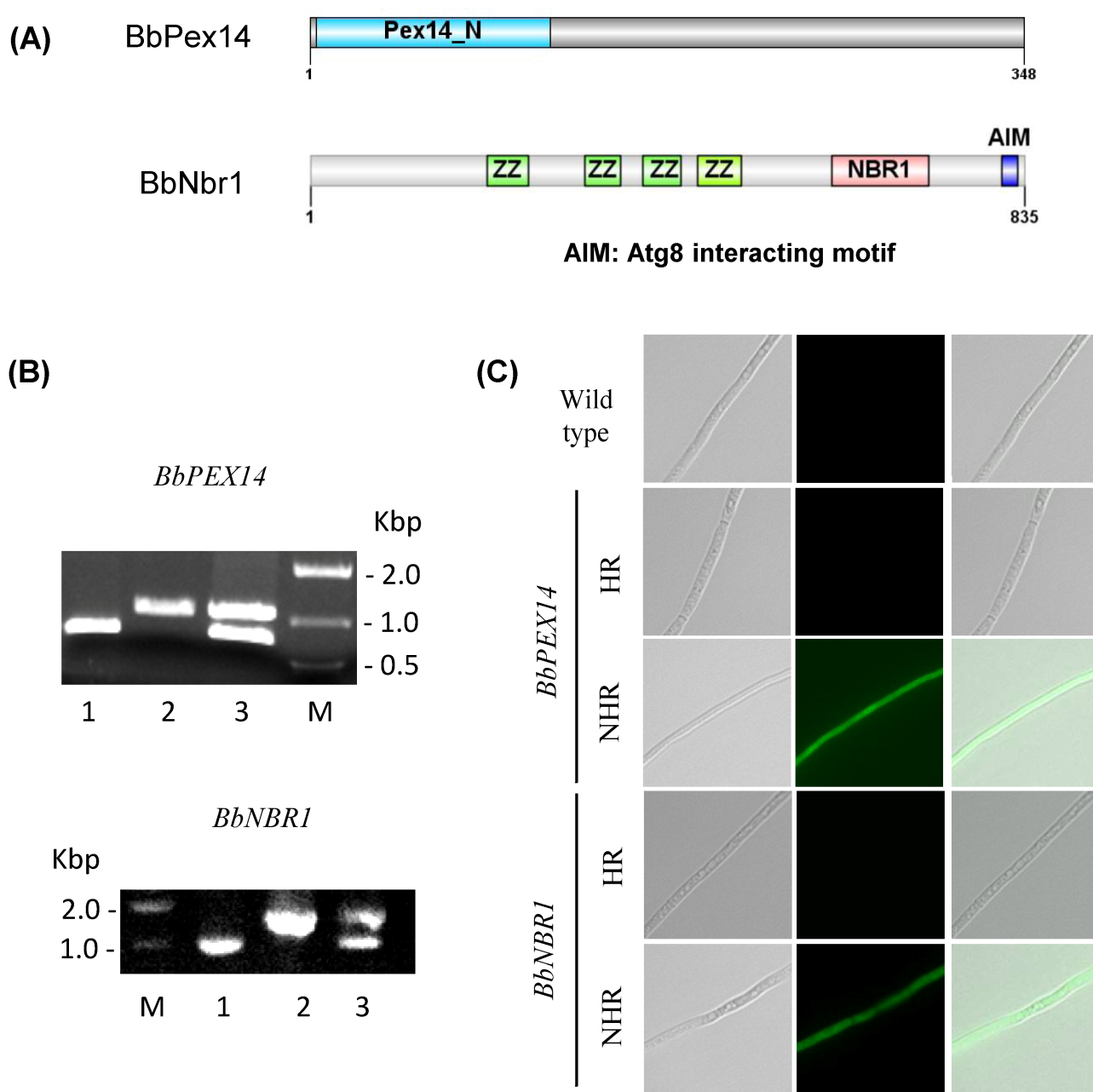


**Figure S2. Imaging the ultra-structure in *B. bassiana* mycelia.** Transmission electron microscopy was used to view peroxisomes (P) in mycelia. Fungal strains were cultured in SDB and the medium with oleic acid as single carbon. There was no significance in peroxisome biogenesis between the wild-type and gene disruption mutant strain.


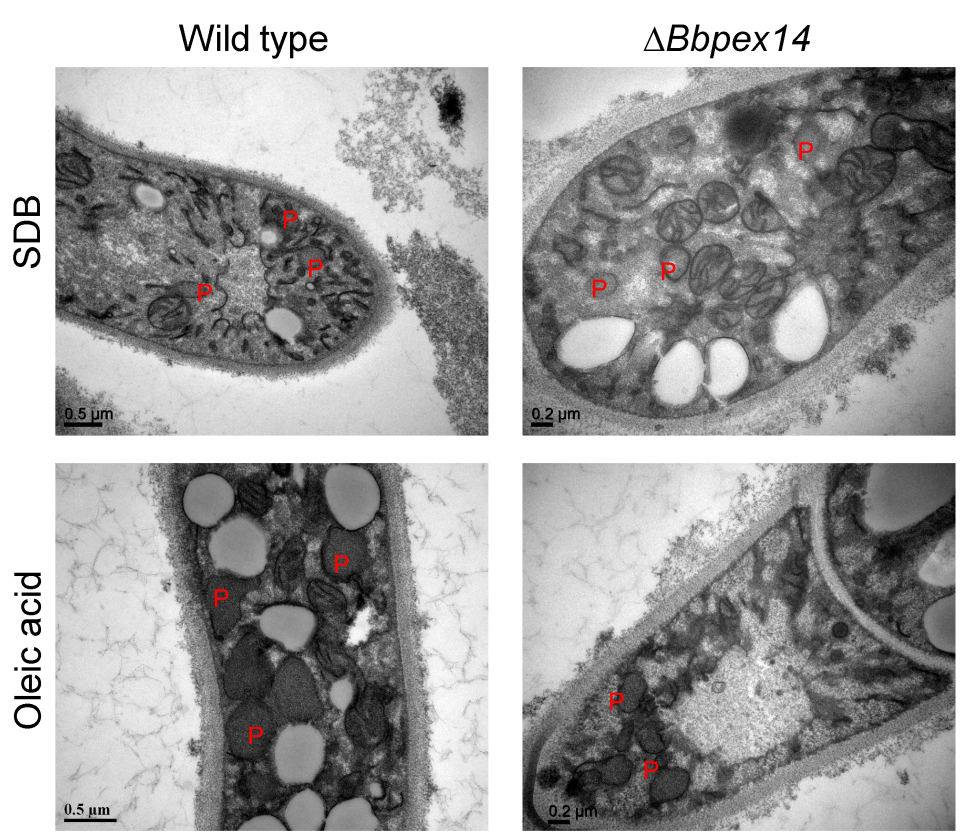


**Figure S3. Assays for fungal growth on various nutrients.** Vegetative growth was examined by culturing fungal strain on the carbon (C) and nitrogen (N) sources for 7 d. (A) Assay for the wild-type, Δ*Bbpex14* and Δ*Bbpex14::BbPEX14* mutant strains. (B) Assay for the wild-type, Δ*Bbnbr1* and Δ*Bbnbr1::BbNBR1* mutant strains. Statistical significance in colony diameter between the wild-type and gene disruption mutant strains was calculated with Student’s *t*-test. *: *P*<0.05.


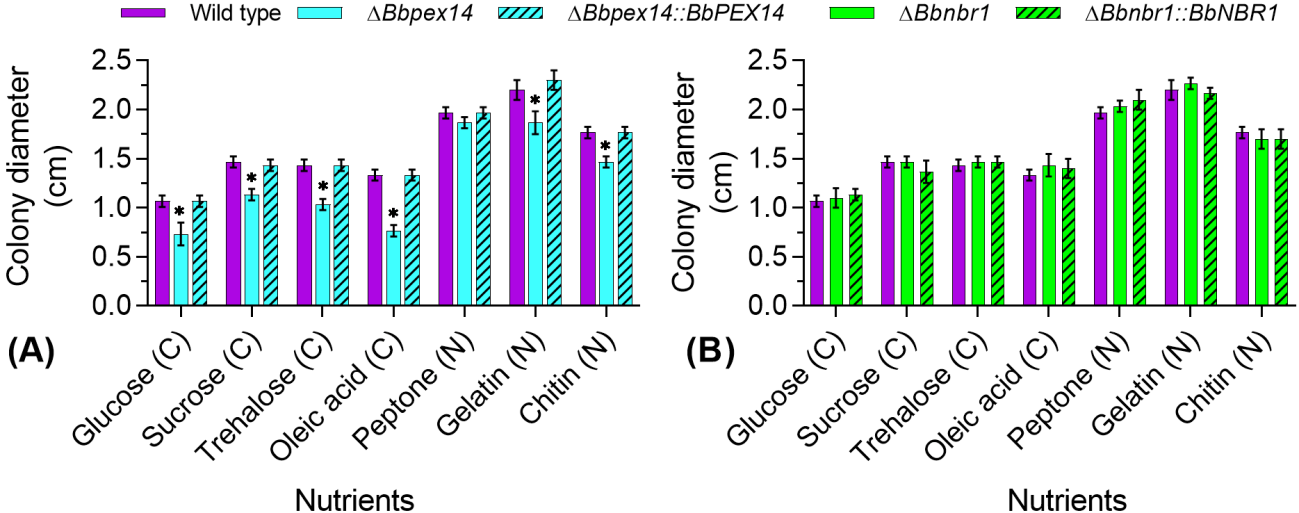


**Figur S4. Protein presence in extracts.** Co-immunoprecipitation was used to determine BbNbr1 interacts with BbPex14 and BbAtg8. *BbPEX14* and *BbATG8* were fused to *Myc*, and *BbNBR1* was fused to *HA*. The wild-type strain was transformed with *BbPEX14-myc* or *BbATG8-myc*. The resultant strain was transformed with *BbNBR1-HA*. The presence of protein in cell exracts was examined with western blot analyses, using actin as endogenous reference. The wild-type strain was used as blank control.


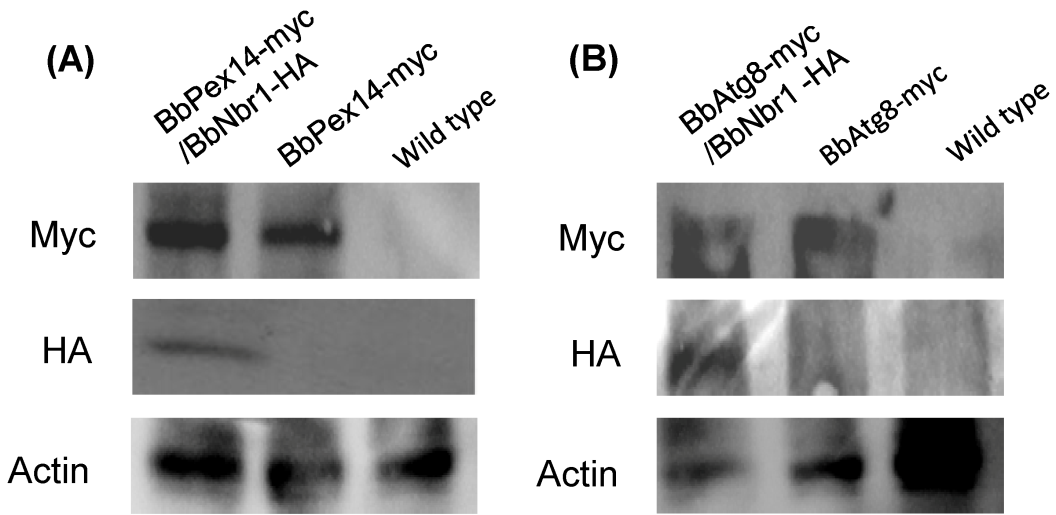

Supplement: Supplemental Material [file KAUO_A_2168337_SM6743.docx]
